# Floods of 18 and 19 November 2016 in Batouri (East Cameroon): Interpretation of the Hydro-Meteorological Parameters and Historical Context of the Post-Event Survey Episode

**DOI:** 10.1155/2019/3814962

**Published:** 2019-01-01

**Authors:** Paulin Sainclair Kouassy Kalédjé, Jules-Rémy Ndam Ngoupayou, Alain Fouépé Takounjou, Mireille Zebsa, Felaniaina Rakotondrabe, Joseph Mvondo Ondoa

**Affiliations:** ^1^Faculty of Science, University of Yaoundé 1, Yaoundé - Cameroon, P.O. Box: 812 Yaoundé, Cameroon; ^2^Hydrological Research Centre, Institute of Geological and Mining Research, Yaoundé, Cameroon; ^3^Research and Innovation Centre, Ministry of Scientific Research and Innovation, Bertoua, Cameroon; ^4^Advanced School of Engineering of Antananarivo, University of Antananarivo, Antananarivo, Madagascar

## Abstract

During the night of November 18 to 19, 2016, many stormy cells are not very mobile organized on the east of the southern plateau of Cameroon and dumped up to 260 mm of rain in 4 hours. Occurring on a relatively saturated soil, these rains caused strong floods of Kadey and Doumé. The floods were particularly damaging in the city of Batouri, where a subdivision was submerged by the Boumbé (tributary of the Sangha) with water heights in the houses reaching 1.75 m, despite the presence of a dam allowing clipping floods upstream of the basin. In this article, we present the results of the analysis of the postevent survey generated on this event with flow rates estimated on 15 sections of ungauged subbasins. These flows are then compared with those obtained from other recent postevent survey and those estimated by various regional estimations. The inventory of heavy rains around Batouri during the period 1970-2016 has led to the revision of current development standards in the region, which seem to underestimate rainfall and infrequent flows.

## 1. Introduction

The intense rains accompanied by flash floods are one of the characteristics of the Equatorial Guinean climate. The extension of urbanized areas in southeastern Cameroon has multiplied homes vulnerable to flooding [1–3]. The collection of data on flash floods is therefore a crucial issue for the definition of flood zones and risk control in urban or peri-urban areas. In recent years, the practice of postevent survey (REX) has grown considerably in Europe and West Africa [4–6] to evaluate flows of these extreme floods. The purpose of the REX analysis is to reconstruct the flows achieved during exceptional events from the flood leashes and the testimony of local residents. These operations have notably helped to reevaluate extreme flows in the Mediterranean and tropical zones, where values greater than 20 m^3^/s/km^2^ over a few km^2^ have frequently been observed [3, 7].

The rain that hits the southeast of Cameroon on 18 and 19 November 2016 had the particularity of reaching in some localities more than 200 mm in 3 hours, with a relatively small spatial extension, a few hundred km^2^ in total, and often only a few dozen square kilometers for the most intense part. This phenomenon is not new in the region, but its location in urbanized areas has led to very significant damage. The characteristics of this episode are described in this article. Flow rates were estimated a posteriori on eighteen (18) sections of ungauged rivers. The values obtained were compared with those of other recent postevent survey from the Southern Cameroon forest. The return periods of the episode were qualified by a regional approach consisting of inventorying similar episodes that occurred in the vicinity of Batouri during the last 50 years. In addition to this article, information on this phenomenon can also be found in other studies conducted by CEREMA (Center for Studies and Expertise on Risks, the Environment, Mobility and Development) (2015) [8], using the same flow estimation method presented here, or by Rey et al. (2016) [9], which describe the environmental consequences of such a hydrometeorological episode.

## 2. Materials and Methods

### 2.1. Characterization of the Area

**Figure 1:**
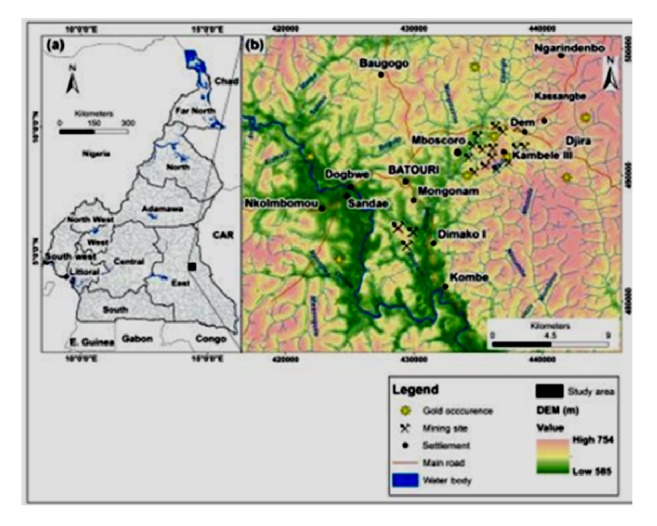
Location of the study site and major rivers.

The area affected by the intense rains of 18 and 19 November 2016 is located in southeastern Cameroon. The most impacted villages were from west to east: Sandae, Dogbwe, Batouri, Mongonam, Mboscoro, Kambélé III, and Dem (Figure 1).

The area is drained by two rivers: Kadey in the West and Boumbé in the East. Kadey has a fairly extensive catchment in the northwest (251 km^2^) but has had little impact during this episode. For the Boumbé, the topographic basin at the Kenzou station is 109 km^2^. The hydrogeological catchment, on a cracked base, feeding the source of the Boumbé covers 372 km^2^ [10]. The rapid recharge area of this catchment is located north of a ridge and was only slightly affected by the event.

The relief, which is a plateau, consists of small hills, and the altitudes vary between 780 and 1000 meters. Metamorphic rocks are largely in the majority but this sector has some karstic outcrops, the latter being located more to the northwest. Land use is made up of scrubland, areas, and urbanized areas dominated by forest and mining structures that have been expanding rapidly in recent decades. The Boumbé subbasin in Kenzou has the following percentages [11]: scrubland 12%, forests 57%, agricultural land 24%, and urban areas 7%. Kadey has fairly similar percentages, but for the two basins, the area affected by the cumulative highs is on the immediate periphery of Batouri; the percentage of urbanized area is higher and is around 25%. For the locality of Batouri, the percentage of urbanized area is close to 100%.

The event of November 18 and 19 occurs in a particular hydrological context, as two episodes of heavy rainfall hit the area the previous month. On October 12th, the stormy rains mainly affect the south-south sector of the eastern region of Cameroon: Bertoua, Mandjou, or Batouri are about 167 mm and Mbang (south of the city) only 383 mm. Then, on October 25, a new episode touches Batouri with 307 mm in 12 hours in Tripano. The southeast Cameroon receives during this episode 142 mm in Bertoua and 122 mm in Mandjou. On 18 November, the soils are very humid, the Kadey cracked basins on the sources of Dem and Lom; Kadey has a flow rate of 0.98 m^3^/s, while the low flow rate before 12 October was 0.07 m^3^/s.

### 2.2. Meteorological Event

On November 18, 2017, the rains started around 9:00 pm (6:00 pm GMT). The results obtained from the weather radar images of France show little mobile storm cells that have been stationed for about 4 hours in the area from Tongo Gadima in the southwest to Tikondi in the northeast, about twenty km, whose width does not exceed 7-8 km. From the analysis of these images, we see that, later in the night, other cells affected the entire Kadey department, between the locality of Mbang and that of Pana, with lower intensities.

**Figure 2:**
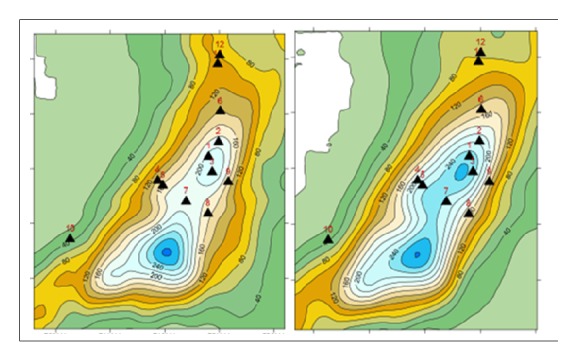
Precipitation casts from November 18, 2016 at 4:00 am GMT to 19 November 2016 at 6:00 am GMT, estimated from the Panthere radar product (left) and OHM-CV reanalysis (right).

Radar images provide an estimate of rainfall and their spatial variability (Figure 2). The Panther radar rain, supplied by Météo-France, has been corrected for rain gauges using a correction factor, constant in space and updated every hour. The spatial resolution is 1 km^2^ and the temporal resolution is 5 mm. The radar rain OHM-CV (Mediterranean Hydrometeorological Observatory Cévennes-Vivarais) corresponds to a reanalysis with a variable correction factor in space, interpolated by kriging. The spatial resolution is 1 km^2^ and the reanalysis is available at a time resolution of 60 minutes.

**Figure 3:**
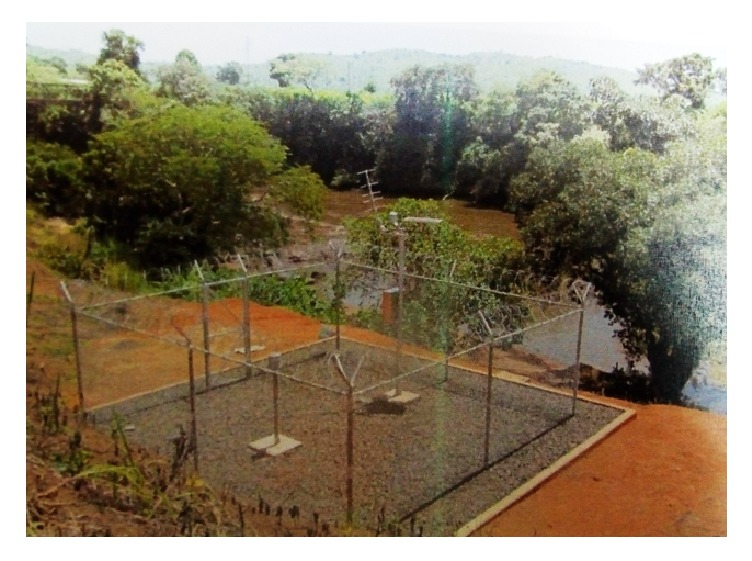
Equipment of the station of Kadey with Batouri in automatic station.

The images differ between Batouri and Kenzou, a sector where Panther totals are underestimated compared to OHM-CV totals. According to the OHM-CV reanalysis, the accumulations are of the order of 280 mm in the commune of Batouri and 265 mm in the commune of Kenzou. According to this same treatment, the areas corresponding to rainfall totals greater than 100, 150, and 200 mm are, respectively, 264, 123, and 67 km^2^. The OHM-CV radar rains are closer to the ground-based rainfall than the Panther radar rains, particularly in Mandjou and Kenzou. It is the consequence of the treatment that spatially interpolates the difference between radar rain and values of the rains obtained by pluviometers on the level of the ground (Figure 3), while the correction of radar rain is spatially uniform in the case of Panther. We will finally retain the OHM-CV rains as a reference.

**Figure 4:**
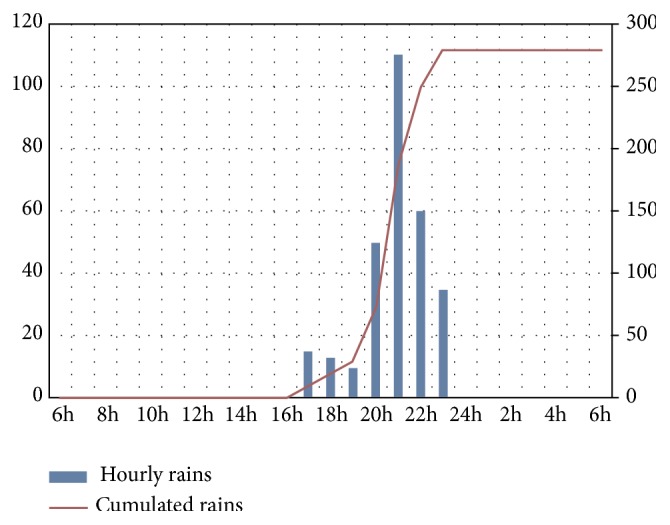
Hyetogram of the rain at the pixel that provided the maximum rain, at Batouri, from 18 November at 6:00 am GMT to 19 November at 6:00 am GMT.

The rainfall hyetograph made from hourly and cumulative rainfall data then shown in Figure 4 for the pixel provided maximum rainfall accumulation in the Kadey department.

## 3. Results and Discussions

### 3.1. Hydrometric Stations of the Measurement Network

Boumbé and Kadey have several hydrometric stations. On the Kadey, the city of Batouri has three stations, but not gauged and whose data are not disseminated. On the Boumbé, two other stations managed by the Regional Direction for the Environment, Planning and Housing (DREAL) are active, but are located downstream of Borgené and are less relevant for this event.

The ordinary flows observed at the Boumbé and Mbang spring are explained by the absence of intense rainfall in the Garou Boulaï-Gado Badjere-Boulembé-Mbang sector, which is the fast recharge area for the Boumbé and Lom springs.

The flow observed on the DREAL threshold of the Boumbé spring is the sum of the outflow of the basin and the flow on a small catchment of 1.02 km^2^. If we subtract the input from the Boumbé spring (11 m^3^/s), the flow corresponding to this basin is exceptional, 36 m^3^/s, i.e., a specific discharge of 35.6 m^3^/s/km^2^. The source of the Boumbé is less than one kilometer from the weather station of Pana.

At the Bertoua station, the Lom flood (479 m^3^/s) is the strongest in the last 10 years of the station's existence. The specific discharge is 5.6 m^3^/s/km^2^ for a catchment with an area of 102 km^2^. Several testimonies concerning the water levels on the road indicate nevertheless that the flood of May 12, 2001, was as important as that of November 19, 2016, at the confluence Boumbé-Kadey.

Further downstream, the flood reaches 499 m^3^/s at Kambélé III on Boumbé, for a catchment area of 115 km^2^, i.e., a specific flow of 4.3 m^3^/s/km^2^. According to the station's records and the flood markings installed by the Hydrological Research Centre, the flood of 19 November 2016 is of the same order of magnitude as those of 1976 (519 m^3^/s), 2005 (487 m^3^/s), and 2003 (440 m^3^/s). The flood of October 2016 reached 344 m^3^/s. The return period of the November 2016 flood seems to be between 10 and 20 years old. This moderate return period is clearly due to the fact that the rainy system has saved significant portions of the catchment area.

On the Kadey, at the Batouri station, in service since 1978, the flood reached 234 m^3^/s and was only exceeded by the 2003 flood (254 m^3^/s). Further upstream, at Dem, over the last 100 years, only the 1957 flood is higher than the 2016 flood mark. In this sector, the return period of the flood is certainly greater than 20 years, but it is difficult to be more precise in the absence of reliable data. It should be kept in mind that the flows on the Bertoua, Batouri, and Kenzou stations are widely extrapolated and that their absolute values are very uncertain.

### 3.2. Flow Estimation by Postevent Survey

To complete the hydrometric information on the ungauged watersheds, the flows were estimated after the floods, by identifying the maximum levels reached and the slopes of the water lines, then applying hydraulic formulas (Gaume and Borga, 2008). In a typical case, it is a question of choosing a rectilinear rectangle without modification of width or break of slope, to measure the cross section at the point considered and the slope of the water line on a few meters or a few tens of meters upstream and downstream of the cross section, finally to apply a Manning-Strickler hydraulic formula to calculate the flow velocity, the roughness coefficient being estimated according to the size of the bed. In other cases, weir-type formulas are applied to water heights estimated at thresholds, or the velocity is estimated by applying the Bernoulli theorem between the upstream and downstream coasts of a structure under load, bridge, or nozzle.

In addition to the two stations located on Boumbé and Lom, flows were estimated for eighteen (18) sections. One of the sections (Batouri) corresponds to the threshold of a reservoir and the flow rate was calculated by a weir formula with a thick threshold, with rectangular section without ridges, in the free regime. The flows of the other sections were estimated from the Manning formula. The calculation method is detailed in other lessons learned from rivers in Europe and West Africa [5, 12, 13]. The 2016 flood surveys and spreadsheets are available in the Ministry of Scientific Research and Innovation database through the Hydrological Research Centre.

The areas of the basins corresponding to these sections were calculated by treatment of the Digital Elevation Models (DEM) 25 m of the National Geographic Institute (NGI). The mean water slides, by correlations and corrections, were calculated for each basin by processing the radar image provided by the OHM-CV.

The flows are expressed in terms of probable flow rates, framed by lower and upper bounds corresponding to the uncertainties on the estimation of these flows. These uncertainties vary from 20 to 50% according to the choice of the section, its regularity, and the legibility of the flood traces. The most important uncertainty factor is often related to the estimation of the Strickler roughness coefficient.

The specific flows vary between 4 and 27 m^3^/s/km^2^. The highest values (Pana, 29.8 m^3^/s/km^2^, and Batouri, 26.3 m^3^/s/km^2^) correspond to the two smaller basins, less than or equal to 1 km^2^. For the other basins, the specific flows vary between 10 and 18 m^3^/s/km^2^, if one considers only the basins having received more than 150 mm of rain. The maximum specific discharge, 18 m^3^/s/km^2^, corresponds to the Batouri catchment, at the heart of the rainy system. The basins with a water slide of less than 150 mm have the lowest specific flows, from 1.4 to 7.5 m^3^/s/km^2^ (Dem and Kambélé III sections upstream of the catchment area of Boumbé), sections of the Kadey incorporating an upstream little watered). Accounting for the impact of pond area on flow abundance, probable flow rates are fairly well organized around

**Figure 5:**
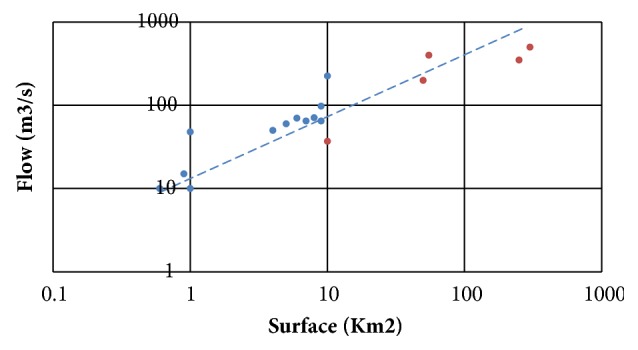
Probable flows based on catchment area.

*Q*_*m*3/*s*_ = 19,96  *X*  *S*_*km*2^0.57^_  (*R*^2^ = 0,81) or *Q*_*m*3/*s*_ = 17,58  *X*  *S*_*km*2^0.76^_  (*R*^2^ = 0,85) if only pools over 150 mm in sizes are considered (Figure 5).

### 3.3. Comparison with Other Exceptional Episodes

Figure 5 compares the results obtained for the event of 18 and 19 November 2016 (considering only basins that received more than 150 mm of rain) with other exceptional episodes since 2000 in southeastern Cameroon. Debit estimates provided by regional flood discharge prediction formulas are also shown:

- ** Bressang-Golossof** (for catchments area between 20 and 400 km^2^):
   
Qm^3^/s  rare = 30 × S(km)0,75
- ** Crupedix:**
   
Outstanding  Qm^3^/s = Q10  years × K2
   
Qm^3^/s(10  years) = S(km2)0,8 × (P(mm)/80)2 × K

The estimated flows are of the same order of magnitude as those observed during other French REX in the Mediterranean region: around Draguignan in the Var in 2010 [5, 14], on the Côte d'Azur around Mandelieu in 2015 [13], and around Abidjan in 2013 [15] and Grand Bassam in 2012 [16]. They remain significantly lower than those estimated during the Gard floods in 2002, at least for basins larger than a few km^2^, or around Lodève in 2015 for nonkarst basins [17]. It is also noted that the estimated feedback values for the episode of November 18 and 19, 2016, exceed the exceptional flows calculated by the Crupedix formula proposed for exceptional flows, while remaining below those given by the Bressang-Golossof rare. It is possible that the decadal rainfall quantile in the area, P10 years = 122 mm, occurring in Crupedix is underestimated.

### 3.4. Estimation of the Return Period of the Rains

The only long series of intraday rains available around Batouri is the Bertoua series, whose statistical analysis was done for the period 1968-2014 by the Hydrological Research Centre in 2017 [18]. The return periods were calculated after adjusting for Weibull law has the observed data for a return period of 50 years; the estimates provided are 49 mm in 30 minutes, 72 mm in 1 hour, and 121 mm in 4 hours.

The Simulation of Hydrograms for the predetermination of floods REGionalized (SHYREG) method [19] based on the synthesis of several hundred series of hourly rainfall and on a regionalization of the parameters from the daily rainfall in the Cameroonian territory provides higher accumulations for durations less than 1 h, but close enough for longer durations: according to National Direction of Meteorology (2014) [20, 21], for the same period of return, fiftieth year, we would have accumulations of the order of 70 mm in 1 hour, 90 mm in 2 hours, and 120 mm in 4 h. For a 100-year return period, the estimates are of the order of 90 mm in 1 h, 100 mm in 2 h, 120 mm in 3 h, and 135 mm in 4 h. According to these estimates, the one-time maximum of the episode of November 18 and 19, 2016, would therefore have a return period well above the centennial.

These estimates can be compared to the inventory of exceptional episodes in the forest massif of southeast Cameroon. Ndam Ngoupayou et al. (2014) [22] state as follows:

- 90 mm in 1 hour on October 27, 1984, in Songloulou.
- 120 mm in 1 hour at Natchigal on 30 September 2001.
- 180 mm in 2 hours at Goura on 25 October 1993.
- 298 mm in 4 h Ebebda and 385 mm in 4 h in Bamendjin on 23 September 1997 (and not in October as the authors have indicated, no remarkable episode having occurred in this month).

These episodes could be mapped from the rainfall data available in the National Direction of Meteorology databases at these dates. For the episode of September 23, 1997, it should be noted that no cumulation available in the National Direction of Meteorology database exceeds 300 mm, the accumulations of Ebebda and Bamendjin from another network of measurements. The 1993 episode seems to be of a different nature from the others, much larger, and more like a Cevennes episode. But an intense rain cell, exceeding locally 200 mm, is also centered on the northeast of Batouri.

**Figure 6:**
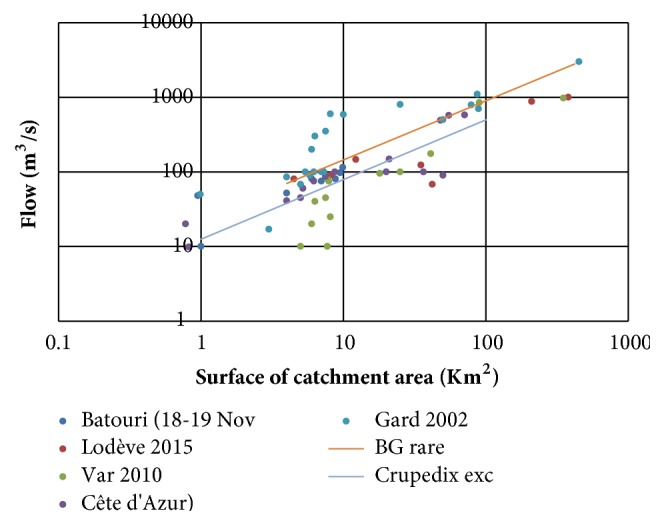
Comparison of flows obtained for different recent postevent survey and for different flood predetermination estimation.

To these episodes are added three more intense episodes (17 September 2007, 29 September 2016, and 18 November 2016) of more than 200 mm in a few hours (3 to 5 hours), for which we have radar rainfall (Figure 6).

Considering only the period 1985-2016, it is thus a total of seven exceptional episodes which were observed on the immediate periphery of Batouri, on an area delimited by Kenzou to the north, Dem to the south, Mbam to the west, and Nbounou to the East. These episodes have in common to have exceeded locally 200 mm in less than 4 hours, which leads to a period of regional return (that is to say, on the area) of less than 7 years for this type of episode. On earlier dates, Chaptal (1934) [23] mentions 227.9 mm fell in 24 hours on September 26 and 27, 1933 (with height of up to 33 mm in 15 minutes and 40 mm in 30 minutes, these intensities cited as being remarkable were only rarely exceeded in 60 years, November 3, 1910, and July 8, 1917), and 233 mm in 7 h on October 11, 1862.

**Figure 7:**
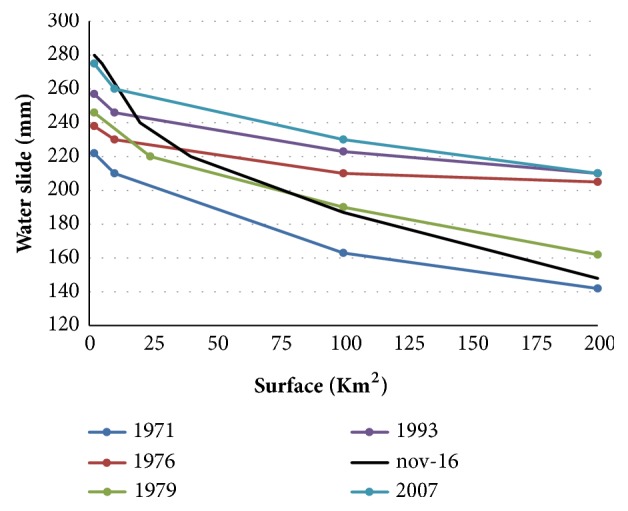
Spatial extension of exceptional episodes exceeding 200 mm in 4 hours.

Areas of water bursts exceeding different thresholds were calculated from interpolated rainfall or radar images (Figure 7). We distinguish the much localized episodes for which the precipitated slide decreases rapidly according to the surface (1971, 2007, and 2016) and those for which it decreases more slowly (1976, 1979, and 1993). For the first group, the areas corresponding to a water level of at least 200 mm vary from 25 to 75 km^2^, while for the second group, they exceed 200 km^2^. The area of the water body exceeding 200 mm can be estimated on average about 100 km^2^.

A very crude reasoning can be used to try to determine the local return period of an exceptional rainfall of at least 200 mm in 4 hours, whose spatial extension is, as we have seen, 100 km^2^ on average. During the period 1985-2016, there are seven episodes of this type, on a fixed area that can be represented by a square of 50 km side. It is inferred that a total of 7 × 100 = 700 km^2^ received a rainfall of at least 200 mm (in 4 hours) during the period considered, i.e., thirty-one (31) years. For each point in the area to be averaged once with a rainfall of more than 200 mm in 4 hours, it would take 2,500/700 more time, or 2,500/700 × 45 = 111 years. The same reasoning, realized for a 150 mm water slide with an average surface area of about 300 km^2^, would lead to estimating the corresponding return period at around 45 years. By comparison, the Simulation of Hydrograms for the predetermination of floods REGionalized (SHYREG) gives this rain a substantially centennial return period. It would therefore tend to overestimate return periods [24–26].

In this calculation, however, there are numerous uncertainties, relative to the estimation of the reference area, the average extent of a given water table, the assumption that the intense rainfall is equiprobable over the reference area, the number of undetected intense episodes, the short length of the reference period with regard to the validity of the statistical analysis, and the spatial abatement allowing to move from the occurrence of a rainfall height to a point to that of an average rainfall of the same height over a given area, which has not been taken into account. In this way, by choosing, for example, a smaller reference area of 900 km^2^ (a square of 30 km on the side), the return period of a water slide of 200 mm in 4 hours would be increased to 60 years and that of a water slide of 150 mm in 4 h to 20 years. It is therefore appropriate to set a fairly broad range for the return period of an episode such as that which affected the Kadey catchment, at least between 60 and 160 years of age, taking into account the uncertainties in the reference area in the method used. In addition, the return period of the October 1998 episode in Pana, whose maximum seems to have reached 420 mm locally in 6:30, was estimated at a little over a hundred years, based on the historical information showing four other comparable episodes over the last 600 years [27]. Davy (1989) [28] also mentions that four showers greater than 200 mm in 6 hours were recorded at the Pana station between 1889 and 1988. These episodes are referring to periods significantly longer than that of the November 2016 rain in Batouri; the estimated return periods cannot be directly compared to those we estimated, but they do not seem to invalidate the range we have proposed [29].

To answer more precisely the question of the frequency of these rains with strong intensities on short durations supposes more important studies. For example, using the recent archives of radar data from 1990 to the present day of the Agency for the Safety of Air Navigation in Africa and Madagascar (ASECNA) coupled with those of the National Direction of Meteorology, it would be possible to study their frequency by taking into account both the area covered by the high intensities and their duration.

## 4. Conclusion

The postevent survey on the floods observed on 18 and 19 November 2016 on the southeast of the South-Cameroon Plateau in Batouri precisely characterized the rains and flows of this meteorological event. Rainfall accumulations of 260 mm in 4 hours have been achieved, and the specific flows are of the order of 10 to 20 m3/s/km2 or more. These flows are of the same order of magnitude or exceeded by those estimated during other recent Cameroonian REX in the forest and humid tropical regions (Congo Basin) on the one hand and on the other hand in several regions: Montpellier-Grabels, Nimes, Var, and Lodève (France); Bouar, Kagabandoro, and Bosangoua (Central Africa Republic); Kano, Adamawa, Taraba, Oyo, Delta Imo Lagos, and Edo (Federal Republic of Nigeria). This type of episode is not so rare in the Kadey and the repetition of these episodes encourages revising the management standards with regard to rare floods at the level of Cameroon but also of the subregions Central Africa and Africa from west. The estimates provided by the statistical analysis of the Batouri local series or by the SHYREG method seem to overestimate the return periods of the rains for intraday durations. Although large uncertainties remain, the return period of rain received by the Kadey Basin in Batouri could be between 60 and 160 years old. In the continuation of this article, a rain-flow model will be proposed to evaluate in the framework of the same episode the role of urbanization or the initial conditions of saturation of soils on the flows, as well as the efficiency of the structures of storage for flood protection.

## Data Availability

The data of the present study are available but can not be published yet, because they are still the property of the Ministry in Charge of Scientific Research and Innovation of Cameroon until December 2019.
